# Emodin Alleviates Hydrogen Peroxide-Induced Inflammation and Oxidative Stress via Mitochondrial Dysfunction by Inhibiting the PI3K/mTOR/GSK3*β* Pathway in Neuroblastoma SH-SY5Y Cells

**DOI:** 10.1155/2020/1562915

**Published:** 2020-08-06

**Authors:** Rui Li, Wenzhou Liu, Li Ou, Feng Gao, Min Li, Liping Wang, Peifeng Wei, Feng Miao

**Affiliations:** ^1^Department of Internal Medicine and Western Medicine, Affiliated Hospital of Shaanxi University of Chinese Medicine, Xianyang, Shaanxi 712046, China; ^2^Department of Traditional Chinese Medicine, Xi'an XD group Hospital, Xi'an, Shaanxi 710077, China; ^3^College of Pharmacy of Shaanxi University of Chinese Medicine, Xianyang, Shaanxi 712046, China; ^4^Affiliated Hospital of Shaanxi University of Chinese Medicine, Xianyang, Shaanxi 712046, China

## Abstract

Emodin is an active monomer extracted from rhubarb root, which has many biological functions, including anti-inflammation, antioxidation, anticancer, and neuroprotection. However, the protective effect of emodin on nerve injury needs to be further elucidated. The purpose of this study is to investigate the effect of emodin on the neuroprotection and the special molecular mechanism. Here, the protective activity of emodin inhibiting H_2_O_2_-induced apoptosis and neuroinflammation as well as its molecular mechanisms was examined using human neuroblastoma cells (SH-SY5Y cells). The results showed that emodin significantly enhanced cell viability, reduced cell apoptosis and LDH release. Simultaneously, emodin downregulated H_2_O_2_-induced inflammatory factors, including IL-6, NO, and TNF-*α*, and alleviated H_2_O_2_-induced oxidative stress and mitochondrial dysfunction in SH-SY5Y cells. In addition, emodin inhibited the activation of the PI3K/mTOR/GSK3*β* signaling pathway. What is more, the PI3K/mTOR/GSK3*β* pathway participated in the protective mechanism of emodin on H_2_O_2_-induced cell damage. Collectively, it suggests that emodin alleviates H_2_O_2_-induced apoptosis and neuroinflammation potentially by regulating the PI3K/mTOR/GSK3*β* signaling pathway.

## 1. Introduction

Neurodegenerative diseases mainly include Parkinson's disease (PD), vascular dementia (VD), and Alzheimer's disease (AD) [1]. According to statistics, the incidence of neurodegenerative diseases is increasing. By 2030, about 65.7 million people will be living with neurodegenerative diseases [2]. Neurodegenerative diseases are mainly caused by oxidative stress, mitochondrial dysfunction, toxins, and inflammation [3–5]. At present, based on the research on the mechanism of neurodegenerative diseases, it is proposed to protect nerve cells from degenerative changes. Therefore, it is urgent to find drugs to protect nerve cells and prevent or treat neurological diseases.

Emodin (1,3,8-trihydroxy-6-methylan-thraquinone), a natural anthraquinone derivative found in the roots and rhizomes of numerous plants such as Polygonum multiflora, rhubarb, and Aloe, is regarded as the most active constituent in Giant Knotweed Rhizome and exerts many potent biological effects, such as anticancer, antimicrobial [6], and anti-inflammatory effects [7]. In the last few years, significant progress has been made in studying the biological effects of emodin at cellular and molecular levels [8]. Studies have demonstrated that emodin is able to induce apoptosis via epithelial-mesenchymal transition (EMT) suppression and caspase-dependent signaling [9–11]. In addition, several studies have revealed that emodin prevents the formation of atherosclerotic plaques [12], reduces neuron cell apoptosis [13], and inhibits glutamate toxicity [14]. However, the specific mechanisms involved require further investigation.

PI3K/mTOR is an important intracellular signaling pathway regulating cell cycle. It regulates cell survival and proliferation by activating ribosome kinase. In addition, the PI3K/mTOR signaling pathway is involved in the regulation of apoptosis [15]. Glycogen synthase kinase-3 (GSK-3) is an evolutionarily conserved serine/threonine kinase, which is ubiquitous in mammalian eukaryotic cells. GSK3*β* acts on various signal proteins and transcription factors, regulating cell differentiation, proliferation, survival, and apoptosis [16]. In the research of neurodegenerative diseases and neuropsychiatric diseases, more and more researchers pay attention to GSK3*β* [17]. It was found that PI3K/mTOR regulated the expression of GSK3*β*, and the PI3K/mTOR/GSK3*β* pathway played an important role in nerve injury [18].

In the mitochondrial-mediated apoptosis pathway, cells are stimulated by apoptotic factors, the mitochondrial membrane potential (ΔΨm) decreases, the permeability of the mitochondrial membrane increases, and then apoptotic factors, such as cyt c and AIF, are released into the cytoplasm [19]. Cyt c interacts with Apaf-1 to form the apoptotic complex. The apoptotic complex recruits and activates caspase-9 and caspase-3, which finally led to apoptosis [20–22]. Studies have revealed that ROS played an important role in the process of mitochondrial-mediated apoptosis, and ROS triggered apoptosis by changing ΔΨm [20, 23].

In the present study, we explored the effect of emodin on the neuroprotection and the special molecular mechanism. Our results demonstrated that emodin alleviates H_2_O_2_-induced apoptosis and neuroinflammation potentially by regulating the PI3K/mTOR/GSK3*β* signaling pathway. It is suggested that emodin may be a potential drug for the treatment of neurodegenerative diseases.

## 2. Materials and Methods

### 2.1. Cell Culture

The SH-SY5Y cells were purchased from the American Type Culture Collection (ATCC, Manassas, VA). The cell was cultured in DMEM, 10% fetal bovine serum (Gibco), 1% glutamine, 100 U/ml penicillin sodium, and 100 *μ*g/ml streptomycin sulfate (Sigma) at 37°C, 5% CO_2_. When the cell number reached 60-70%, it was washed with PBS for standby.

### 2.2. Reagents

The SH-SY5Y (San Diego, CA, USA); GAPDH, anti-PI3K, anti-mTOR, anti-COX-2, anti-iNOS, and anti-GSK3*β* (Wuhan Sanying Biotechnology Co., Ltd., Wuhan, China); DMEM (Gibco, Carlsbad, CA, USA); RNA extraction kit, reverse transcription kit, and RT-PCR kit (Invitrogen, Carlsbad, CA, USA); primer synthesis (Takara, Dalian, China); and protein quantitative kit and cell lysate (Biyuntian Biotechnology Research Institute, Nantong City, China) were purchased. ELISA kits were purchased from Westang Technology Ltd. (Shanghai, China).

### 2.3. Transfection

SH-SY5Y cells were inoculated with 1 × 10^5^ cells/well on the 24-well plate. When the cell growth convergence rate reached about 60%, the PI3K inhibitor and GSK3*β* inhibitor were transfected into SH-SY5Y cells according to the instructions of Lipofectamine™ 3000 transfection reagent. The final transfection concentration was 50 ng/l. After 6 h of culture in 37°C and 5% CO_2_ incubator, the fresh medium was replaced for further culture. The culture was finished later 48 h, and the cells of each group were collected for follow-up experiment.

### 2.4. Cell Viability Assay

SH-SY5Y cells were inoculated with 1 × 10^5^ cells/well on the 96-well plate. The cells were incubated with different concentrations of emodin for 24 h. MTT in 0.1 mm PBS was added to each well and cultured at 37°C for 4 h. Add extraction buffer and then culture in 37°C and 5% CO_2_ incubator for 24 h; OD value at 590 nm was determined by enzyme-labeling instrument. The above experiments were repeated three times, and the average value was obtained.

### 2.5. Cell Apoptosis Analysis

SH-SY5Y cells were treated with emodin in 6-well plates and incubated for 24 h. Finally, the cells were collected and incubated with Annexin-V and propidium iodide (PI) for 20 min. The incidence of apoptosis was evaluated by a flow cytometer (FCM).

### 2.6. ELISA

The TNF-*α* (BioSite, Paris, France), NO (BioSite, Paris, France), and IL-6 (BioSite, Paris, France) concentrations in the supernatants were detected using corresponding ELISA kits according to the instructions. The absorbance was measured at 450 nm using a microplate reader. The above experiments were repeated three times, and the average value was obtained.

### 2.7. Measurement of NAD^+^

Intracellular NAD^+^ levels were measured using the EnzyChrom NAD^+^/NADH assay kit (BioAssay Systems, Hayward, CA, USA). SH-SY5Y cells were washed with PBS and then lysed with the supplied NAD extraction buffer. NAD^+^ was extracted from the lysate according to the manufacturer's protocol. The measurement of NAD^+^ is based on an alcohol dehydrogenase cycling reaction. The change in absorbance at 565 nm for 15 min at room temperature was measured.

### 2.8. Measurement of ATP

SH-SY5Y cells were treated with emodin for 24 h, and then, the culture medium was removed, stored, and replaced by HEPES buffer. After washing, the initially stored culture medium was added to cells for 1 h. The cells were lysed with 10 mM Tris-HCl (pH 7.8), and ATP content was determined using a quantitative bioluminescent assay (Sigma, St. Louis, MO, USA) according to the instructions of the manufacturer and an iMark microplate absorbance reader (Bio-Rad, Hercules, CA, USA).

### 2.9. Measurement of ROS by FCM

Briefly, SH-SY5Y cells were incubated in 6-well plates overnight. The cells were collected and incubated with ROS indicator DCFH-DA (10 *μ*M) in PBS for 30 min at 37°C. The fluorescence was analyzed using an Accuri C6 plus flow cytometer (BD Biosciences, CA).

### 2.10. Evaluation of ΔΨm

SH-SY5Y cells were seeded in 6-well plates at a density of 1 × 10^5^ cells/well. Cells were collected and stained with 10 *μ*M JC-1 solution in PBS for 30 min at 37°C. Then, the images were acquired by the flow cytometer. When the mitochondrial membrane potential is increased, JC-1 will gather in the mitochondrial matrix to form a polymer, which produces red fluorescence, showing double-positive FL1 and FL2 in the flow pattern; when the mitochondrial membrane potential is decreased, JC-1 cannot gather in the mitochondrial matrix, and JC-1 is a monomer, which produces green fluorescence. The change of the mitochondrial membrane potential is shown in the different fluorescent colors [24].

### 2.11. RT-qPCR

Total RNA samples from the SH-SY5Y cells were isolated using TRIzol^®^ reagent (Invitrogen, Carlsbad, CA, USA), and NanoDrop 2000 instrument was used to detect the RNA concentration of samples. Then, the total RNA was retrotranscribed into cDNA according to the instructions of the reverse transcription kit. According to the instructions of the qPCR kit, configure the corresponding system and set up 3 multiple wells for each group. Use the TB Green™ Premix Ex Taq II kit for qPCR. The conditions of qPCR were as follows: 95°C 30 s (1 cycle), 95°C 5 s, and 60°C 30 s (35 cycles). The data were analyzed by the 2^–*ΔΔ*CT^ method with GAPDH as a normalizing gene. The experiment was repeated three times independently.

### 2.12. Western Blotting

The cells were inoculated into 6-well plates, 1 × 10^5^ cells/well, and the supernatant was removed 24 h later. The plasmid was transfected and the cells were collected 48 h. The protein extracted from the cells was collected. Cells were lysed with improved RIPA buffer (Sigma-Aldrich), and the protein content was measured by Bradford reagent (Thermo Scientific). The extracted protein (50 *μ*g) was separated from denatured polyacrylamide gel and then transferred to PVDF membrane (microporous), sealed with 5% skim milk (HiMedia). Then, the Enhanced Laboratories (ECL) darkroom development, Bio-Rad Laboratories (California, USA) scan record, and anti-GAPDF as internal reference were used for analysis and comparison.

### 2.13. Statistical Analyses

Data are represented as mean ± SD, and each experiment was performed in triplicate in this study. One-way or two-way ANOVA and Student's unpaired *t*-test were used to analyze statistical significance. All statistical analyses were performed by SPSS 20.0 software (SPSS, Inc., Chicago, IL, USA). *P* value < 0.05 were considered to be significant.

## 3. Results

### 3.1. Emodin Alleviates Apoptosis of H_2_O_2_-Induced SH-SY5Y Cells

**Figure 1:**
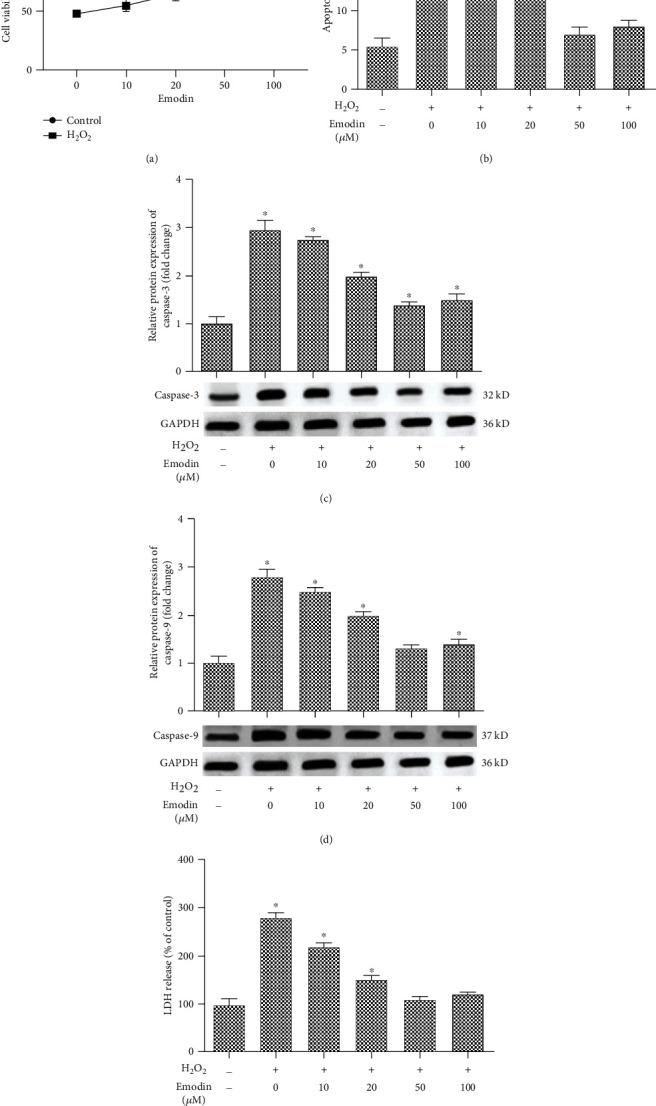
Emodin alleviates apoptosis of H_2_O_2_-induced neuroblastoma cell SH-SY5Y. SH-SY5Y cells were treated with emodin of different concentrations (0, 10, 20, 50, and 100 *μ*M) for 24 h and with H_2_O_2_ (200 *μ*M) for 1 h. The experiment was divided into the control, 0, 10, 20, 50, and 100 *μ*M groups. (a) The cell viability rate was determined using the MTT. (b) The apoptosis of SH-SY5Y was determined using V-FITC/PI. (c) The protein expression of caspase-3. (d) The protein expression of caspase-9. (e) LDH release was measured in the same experimental conditions. “∗” means compared with the control group at *P* < 0.05. GAPDH was used as an invariant internal control for calculating protein fold changes.

To investigate whether emodin exerts protective effects against H_2_O_2_-induced apoptosis, SH-SY5Y cells were treated with various concentrations of emodin (10, 20, 50, and 100 *μ*M) for 24 h, followed by exposing to H_2_O_2_ (200 *μ*M) for 1 h. As shown in Figure 1(a), the cell viability of H_2_O_2_-induced SH-SY5Y cells was significantly increased with the increase of emodin concentration, while there was no significant difference in cell viability when different concentrations of emodin was used alone (Figure 1(a)). More importantly, emodin significantly reduced the apoptosis of SH-SY5Y cells in different concentrations (Figure 1(b)). What is more, the expression of caspese-3 and caspese-9 was significantly decreased compared with the H_2_O_2_-induced group, and in a dose-dependent manner (Figures 1(c) and 1(d)). In addition, emodin treatment significantly reduced LDH release (Figure 1(e)). According to the above experimental results, 50 *μ*M of emodin was used in the following experiments.

### 3.2. Emodin Alleviates the Inflammation of H_2_O_2_-Induced SH-SY5Y Cells

**Figure 2:**
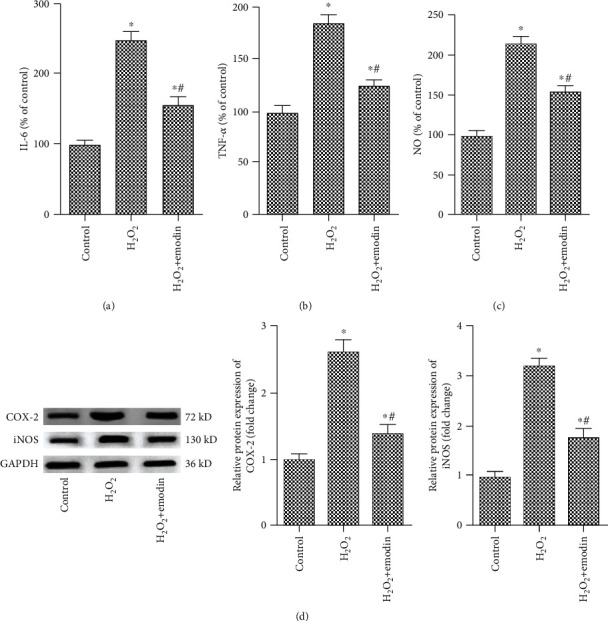
Emodin alleviates the inflammation of H_2_O_2_-induced neuroblastoma cell SH-SY5Y. SH-SY5Y cells were treated with emodin at a concentration of 50 *μ*M for 24 h and with H_2_O_2_ (200 *μ*M) for 1 h. The experiment was divided into the control group, H_2_O_2_ group, and H_2_O_2_+emodin group. The levels of proinflammatory factors were detected by ELISA and Western blot. (a–c) The level of IL-6, TNF-*α*, and NO in the control group, H_2_O_2_ group, and H_2_O_2_+emodin group. (d) The protein expression of COX-2 and iNOS in the control group, H_2_O_2_ group, and H_2_O_2_+emodin group. “∗” means compared with the control group at *P* < 0.05, and “#” means compared with the H_2_O_2_ group at *P* < 0.05. GAPDH was used as an invariant internal control for calculating protein fold changes.

SH-SY5Y cells were treated with concentrations of emodin (50 *μ*M) for 24 h, followed by exposing to H_2_O_2_ (200 *μ*M) for 1 h, and the levels of proinflammatory factors were detected by ELISA and Western blot. Our results showed that the levels of IL-6, TNF-*α*, and NO were significantly increased after H_2_O_2_ treatment compared with the untreated group, while emodin (50 *μ*M) significantly decreased the level of IL-6, NO, and TNF-*α* (Figures 2(a)–2(c)). In addition, the expression of COX-2 and iNOS was increased after H_2_O_2_ treatment, while emodin (50 *μ*M) decreased the expression of COX-2 and iNOS (Figure 2(d)).

### 3.3. Emodin Alleviates Mitochondrial Dysfunction and Oxidative Stress of H_2_O_2_-Induced SH-SY5Y Cells

**Figure 3:**
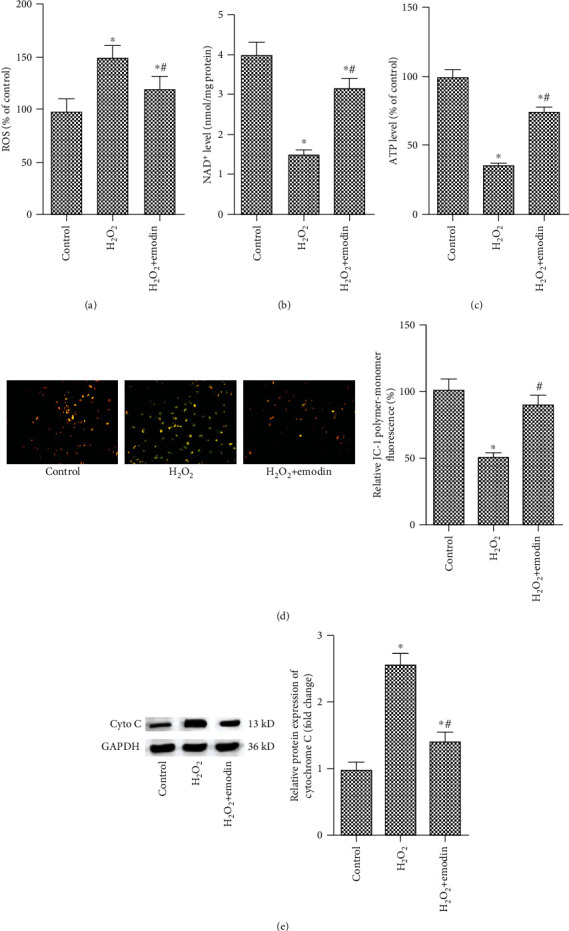
Emodin alleviated mitochondrial dysfunction and oxidative stress of H_2_O_2_-induced neuroblastoma cell SH-SY5Y. SH-SY5Y cells were treated with emodin at a concentration of 50 *μ*M for 24 h and with H_2_O_2_ (200 *μ*M) for 1 h. The experiment was divided into the control group, H_2_O_2_ group, and H_2_O_2_+emodin group. (a) The ROS level in the control group, H_2_O_2_ group, and H_2_O_2_+emodin group. (b) The NAD^+^ level in the control group, H_2_O_2_ group, and H_2_O_2_+emodin group. (c) The ATP level in the control group, H_2_O_2_ group, and H_2_O_2_+emodin group. (d) Mitochondrial membrane potential measured by JC-1. (e) The protein expression of cytochrome c in the control group, H_2_O_2_ group, and H_2_O_2_+emodin group. “∗” means compared with the untreated group at *P* < 0.05, and “#” means compared with the H_2_O_2_ group at *P* < 0.05. GAPDH was used as an invariant internal control for calculating protein fold changes.

Mitochondrial dysfunction and oxidative stress are the critical causes of apoptosis [25]. Therefore, we examined whether emodin affects the mitochondrial function and the level of oxidative factors in SH-SY5Y cells. The results showed that the levels of ROS were significantly increased after H_2_O_2_ treatment compared with the untreated group, while emodin (50 *μ*M) significantly decreased the level of ROS (Figure 3(a)). However, the levels of NAD^+^ and ATP were significantly decreased when exposed to H_2_O_2_, while emodin (50 *μ*M) significantly increased the level of NAD^+^ and ATP (Figures 3(b) and 3(c)). What is more, emodin (50 *μ*M) treatment significantly prevented the damage of mitochondrial function caused by H_2_O_2_ exposure (Figure 3(d)). Additionally, H_2_O_2_ significantly increased the expression of cytochrome c, and emodin (50 *μ*M) reversed the increased of cytochrome c expression induced by H_2_O_2_ (Figure 3(e)).

### 3.4. Emodin Inhibits the Activation of the PI3K/mTOR/GSK3*β* Signaling Pathway Induced by H_2_O_2_

**Figure 4:**
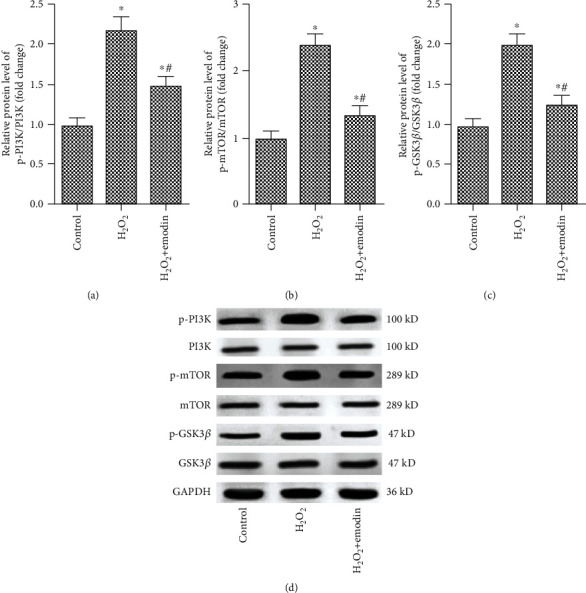
Emodin inhibits the activation of the PI3K/mTOR/GSK3*β* pathway induced by H_2_O_2_. SH-SY5Y cells were treated with emodin at a concentration of 50 *μ*M for 24 h and with H_2_O_2_ (200 *μ*M) for 1 h. The experiment was divided into the control group, H_2_O_2_ group, and H_2_O_2_+emodin group. (a) Western blot was performed to confirm the protein expression levels of PI3K, p-PI3K, mTOR, p-TOR, GSK3*β*, and p-GSK3*β*. (b) The protein expression of p-PI3K/PI3K in the control group, H_2_O_2_ group, and H_2_O_2_+emodin group. (c) The protein expression of p-mTOR/mTOR in the control group, H_2_O_2_ group, and H_2_O_2_+emodin group. (d) The protein expression of p-GSK3*β*/GSK3*β* in the control group, H_2_O_2_ group, and H_2_O_2_+emodin group. “∗” means compared with the untreated group at *P* < 0.05, and “#” means compared with the H_2_O_2_ group at *P* < 0.05. GAPDH was used as an invariant internal control for calculating protein fold changes.

In order to further explore the protective mechanism of emodin on SH-SY5Y cells, we investigated the effect of emodin on the expression of the PI3K/mTOR/GSK3*β* signaling pathway-related protein. As shown in Figure 4, H_2_O_2_ treatment significantly increased the expression of p-PI3K, p-mTOR, and p-GSK3*β*, while emodin (50 *μ*M) significantly reduced the expression of p-PI3K, p-mTOR, and p-GSK3*β*, indicating that emodin (50 *μ*M) inhibited the activation of the PI3K/mTOR/GSK3*β* pathway induced by H_2_O_2_ (Figure 4).

### 3.5. PI3K/mTOR/GSK3*β* Pathway Participates in the Protective Mechanism of Emodin on H_2_O_2_-Induced Cell Damage

**Figure 5:**
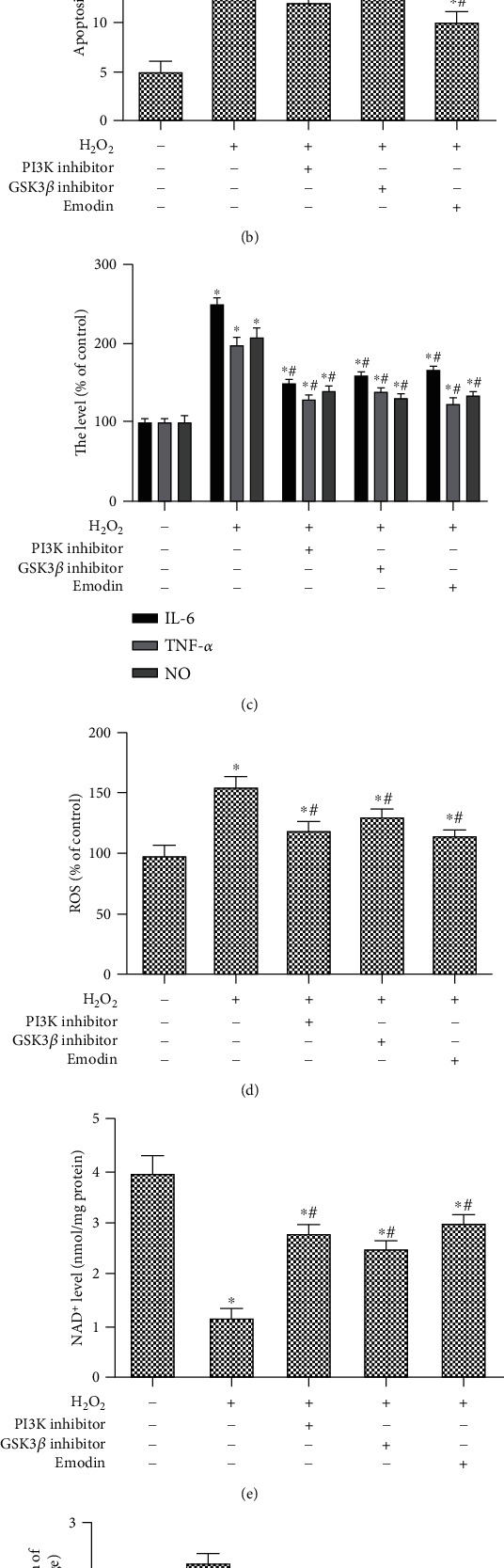
PI3K/mTOR/GSK3*β* pathway participates in the protective mechanism of emodin on H_2_O_2_-induced cell damage. SH-SY5Y cells were transfected with the PI3K inhibitor or GSK3*β* inhibitor, respectively, and treated with emodin at a concentration of 50 *μ*M for 24 h and with H_2_O_2_ (200 *μ*M) for 1 h. (a) The cell viability rate was determined using the MTT in each group. (b) The apoptosis rate (%) of SH-SY5Y in each group. (c) The level of IL-6, TNF-*α*, and NO of SH-SY5Y in each group. (d) The ROS level of SH-SY5Y in each group. (e) The NAD^+^ level of SH-SY5Y in each group. (f) The protein expression of cytochrome c of SH-SY5Y in each group. “∗” means compared with the untreated group at *P* < 0.05, and “#” means compared with the H_2_O_2_ group at *P* < 0.05. GAPDH was used as an invariant internal control for calculating protein fold changes.

SH-SY5Y cells were transfected with the PI3K/mTOR inhibitor and GSK3*β* inhibitor or treated with emodin, respectively. Our findings displayed that the PI3K/mTOR inhibitor, GSK3*β* inhibitor, and emodin (50 *μ*M) all significantly alleviated H_2_O_2_-induced apoptosis and cell viability (Figures 5(a) and 5(b)). In addition, the levels of IL-6, TNF-*α*, and NO were significantly increased after treatment with the PI3K/mTOR inhibitor, GSK3*β* inhibitor, and emodin (50 *μ*M) (Figure 5(c)). More importantly, the PI3K/mTOR inhibitor, GSK3*β* inhibitor, and emodin (50 *μ*M) all significantly protected cells from H_2_O_2_-induced oxidative stress, inhibited the expression of cytochrome c, and improved mitochondrial function (Figures 5(d)–5(f)).

## 4. Discussion

Recent studies have shown that SH-SY5Y cells have been used in many neurodegenerative disease models, including PD, VD, and AD [26–28]. In the present research, we suggested that emodin was found to alleviate H_2_O_2_-induced apoptosis of SH-SY5Y cells. Furthermore, emodin alleviated H_2_O_2_-induced neuroinflammation by regulating the PI3K/mTOR/GSK3*β* pathway. Therefore, emodin may be a new treatment for neurodegenerative disease.

Emodin plays an important role in protecting neurons from injury [29]. It is reported that emodin inhibited the excitatory postsynaptic potential and blocked the transmission of stimulation signals by reducing the release of glutamate [30]. In addition, Liu et al. [31] suggested that emodin inhibited the production of neurotoxin and cell apoptosis by inhibiting the activation of the AMPK signaling pathway induced by Zn^2+^. Likewise, emodin is involved in the antineuroinflammatory process of LPS-stimulated microglia by activating the AMPK/Nrf2 signaling pathway, which suggests that emodin can be used as a natural antineuroinflammatory agent. In the present research, our results show that emodin not only protects cells from apoptosis; on the other hand, emodin inhibits the production of inflammatory factors in cells and alleviates H_2_O_2_-induced mitochondrial dysfunction and oxidative stress. This is consistent with the previous research conclusion, indicating that emodin does play an important role in the development of nerve injury and neuroinflammation. Therefore, emodin may be a potential drug for the treatment of neuroinflammatory diseases.

Oxidative stress is considered to be associated with various diseases, including Alzheimer's disease, amyotrophic lateral sclerosis, Friedreich's ataxia, Huntington's disease, and Parkinson's disease [32]. Mitochondria are one of the main sources of oxidative stress (ROS), because it utilizes oxygen to produce energy. Studies have shown that ROS is a crucial factor and plays an important role in the process of the mitochondrial-mediated apoptosis pathway via altering the mitochondrial membrane potential, which made mitochondrial dysfunction and then could disturb the mitochondrial-mediated apoptosis [5, 20, 33]. What is more, excessive stimulation of NADH leads to excessive accumulation of ROS, oxidative stress, and damage of cell structure, protein, and DNA [23]. Interestingly, our finding revealed emodin reduced ROS accumulation, maintained NAD^+^ and ATP levels, balanced mitochondrial membrane potential (ΔΨm), and protected cells from apoptosis and oxidative stress. These results indicate that emodin can protect neurons from damage by alleviating oxidative stress.

Herein, we subsequently examined the mechanisms of emodin-protected cells from apoptosis and oxidative stress. We found that emodin reduced the expression of the PI3K/mTOR/GSK3*β* pathway-related protein in H_2_O_2_-induced SH-SY5Y cells, indicating that emodin inhibited the activation of the PI3K/mTOR/GSK3*β* pathway. It has been reported that the PI3K/mTOR/GSK3*β* signaling pathway participates in the regulation of various neurodegenerative diseases. Activation and inflammatory responses of SH-SY5Y cells are usually associated with the PI3K/mTOR/GSK3*β* signaling pathways, which in turn trigger a range of neurological diseases, such as hypothalamic inflammation [34], AD [35], and PD [36]. Yang et al. [18] reported that MALAT1 inhibited the proliferation of SH-SY5Y cells, promoted apoptosis, induced the production of A*β*25-35, and induced Alzheimer's disease by inhibiting the activation of the PI3K/mTOR/GSK3*β* pathway. Analogously, emodin induced axon production of Neuro2a cells by regulating the PI3K/mTOR/GSK3*β* pathway. Many literatures have reported that PI3K/Akt activation promotes the activation of GSK3*β* and then induces various diseases, including cancer, neurological diseases, and inflammatory diseases [18]. Likewise, we found that the PI3K also activated GSK3*β*, and the PI3K/mTOR inhibitor and GSK3*β* inhibitor alleviated H_2_O_2_-induced mitochondrial dysfunction and oxidative stress-mediated apoptosis. It is suggested that the PI3K/mTOR/GSK3*β* pathway is involved in the regulation of apoptosis and inflammation of SH-SY5Y cells. Therefore, inhibiting the PI3K/mTOR/GSK3*β* pathway may be an effective treatment for neurodegenerative diseases.

In summary, we explored the effect of emodin on the process of neuroprotection and the special molecular mechanism. We found that emodin alleviates H_2_O_2_-induced apoptosis, neuroinflammation, mitochondrial dysfunction, and oxidative stress via regulating the PI3K/mTOR/GSK3*β* signaling pathway. The results suggest that emodin may be a potential drug for the treatment of neurodegenerative diseases.

## Data Availability

The data used to support the findings of this study are available from the corresponding authors upon request.
